# Soluble CD40 Ligand Stimulates CD40-Dependent Activation of the β2 Integrin Mac-1 and Protein Kinase C Zeda (PKCζ) in Neutrophils: Implications for Neutrophil-Platelet Interactions and Neutrophil Oxidative Burst

**DOI:** 10.1371/journal.pone.0064631

**Published:** 2013-06-06

**Authors:** Rong Jin, Shiyong Yu, Zifang Song, Xiaolei Zhu, Cuiping Wang, Jinchuan Yan, Fusheng Wu, Anil Nanda, D. Neil Granger, Guohong Li

**Affiliations:** 1 Vascular Biology and Stroke Research Laboratory, Department of Neurosurgery, Louisiana State University Health Science Center in Shreveport, Shreveport, Louisiana, United States of America; 2 Department of Physiology, Louisiana State University Health Science Center in Shreveport, Shreveport, Louisiana, United States of America; 3 Institute of Cardiovascular Diseases, Xinqiao Hospital, Third Military Medical University, Chongqing, China; 4 Department of Cardiology, The Affiliated Hospital of Jiangsu University, Jiangsu, Zhenjiang, China; 5 Department of Surgical Oncology, The First Affiliated Hospital, Zhejiang University, Hangzhou, China; Northwestern University Feinberg School of Medicine, United States of America

## Abstract

Recent work has revealed an essential involvement of soluble CD40L (sCD40L) in inflammation and vascular disease. Activated platelets are the major source of sCD40L, which has been implicated in platelet and leukocyte activation, although its exact functional impact on leukocyte-platelet interactions and the underlying mechanisms remain undefined. We aimed to determine the impact and the mechanisms of sCD40L on neutrophils. We studied neutrophil interactions with activated, surface-adherent platelets as a model for leukocyte recruitment to the sites of injury. Our data show that CD40L contributes to neutrophil firm adhesion to and transmigration across activated surface-adherent platelets, possibly through two potential mechanisms. One involves the direct interaction of ligand-receptor (CD40L-CD40), i.e., platelet surface CD40L interaction with neutrophil CD40; another involves an indirect mechanism, i.e. soluble CD40L stimulates activation of the leukocyte-specific β2 integrin Mac-1 in neutrophils and thereby further promotes neutrophil adhesion and migration. Activation of the integrin Mac-1 is known to be critical for mediating neutrophil adhesion and migration. sCD40L activated Mac-1 in neutrophils and enhanced neutrophil-platelet interactions in wild-type neutrophils, but failed to elicit such responses in CD40-deficient neutrophils. Furthermore, our data show that the protein kinase C zeta (PKCζ) is critically required for sCD40L-induced Mac-1 activation and neutrophil adhesive function. sCD40L strongly stimulated the focal clustering of Mac-1 (CD11b) and the colocalization of Mac-1 with PKCζ in wild-type neutrophils, but had minimal effect in CD40-deficient neutrophils. Blocking PKCζ completely inhibited sCD40L-induced neutrophil firm adhesion. Moreover, sCD40L strongly stimulates neutrophil oxidative burst via CD40-dependent activation of PI3K/NF-KB, but independent of Mac-1 and PKCζ. These findings may contribute to a better understanding of the underlying mechanisms by which sCD40L/CD40 pathway contributes to inflammation and vascular diseases.

## Introduction

Platelet activation and leukocyte-platelet interactions play an important role in the pathogenesis of vascular disease including atherosclerosis and restenosis [1]–[3]. Experimental studies have demonstrated that after arterial denudation injury platelet deposition precedes leukocyte accumulation at sites of injury, and early recruitment of leukocytes to the sites of injury is likely mediated through leukocyte-platelet adhesive interactions followed by leukocyte transmigration through the surface-adherent platelet monolayer, resulting in leukocyte infiltration into vessel wall [4], [5]. It is thus proposed that platelets deposited at injured vessel wall form an adhesive surface that promotes leukocyte recruitment through direct interaction of ligand-receptor pairs between platelets and leukocytes, although the mechanism of the interaction has not been completely clarified [2]. Although much attention has been paid to the role of monocytes/macrophages in restenosis, activation and recruitment of neutrophils are also regarded to play a key role in the mechanism of restenosis, both clinically [6]–[10] and in experimental studies [11], [12]. Neutrophils are the first cells to be recruited to the site of inflammation and injury [2]. Infiltrating neutrophils contribute to the pathogenesis of restenosis possibly through their ability to generate an oxidative burst and release metalloproteinases [2]. For example, stent-induced neutrophil activation is associated with an oxidative burst in the post-stent inflammatory process, possibly leading to restenosis [8].

The CD40 ligand (CD40L, CD154), a member of the TNF superfamily, and its receptor CD40 have been implicated in inflammation and the pathophysiology of various inflammatory diseases [13]. Activated platelets not only express CD40L on their surface, but also constitute the major source of soluble CD40L (sCD40L), accounting for >95% of sCD40L in the blood [14], [15]. Recent work has revealed an essential involvement of sCD40L and its receptor CD40 in atherosclerosis and restenosis. Elevated plasma levels of sCD40L in patients have now emerged as a reliable predictor of cardiovascular events, such as atherosclerotic plaque rupture and acute coronary syndromes [13]. We and others have demonstrated that elevated sCD40L increases leukocyte recruitment and neointimal formation after arterial injury [16], [17]. Whereas disruption of sCD40L/CD40 has been shown to inhibit atherosclerosis and neointima formation after vascular injury, the underlying mechanisms have not yet been completely clarified. In addition to its classic counter receptor CD40, CD40L can also directly interact with several other receptors including the leukocyte-specific β2 integrin Mac-1 (CD11b/CD18) [13]. Neutrophils are known to express both CD40 and Mac-1 [16], [18]. This study aimed to determine the impact and mechanisms of sCD40L on neutrophils, with focus on platelet-neutrophil interactions and neutrophil oxidative stress, through interaction with its counterreceptor CD40 and/or integrin Mac-1 in vitro.

## Materials and Methods

### Ethics statement

Platelets and leukocytes were prepared from C57Bl6 (wild-type [WT]), CD40^−/−^, CD40L^−/−^, and MAC-1^−/−^ knockout mice. All the mice (on a C57Bl6 background) were obtained from the Jackson Laboratory (Bar Harbor, ME). The study design was approved by the local Ethic Committee of the LSU Health Sciences Center, Shreveport, Louisiana.

### Reagents and antibodies

Recombinant mouse CD40 ligand (rm-CD40L) was purchased from R&D Systems (Minneapolis, MN). The rabbit anti-PKCζ polyclonal antibody and the rat anti-mouse Mac-1 (CD11b, clone M1/70) monoclonal antibodies were obtained from Cell Signaling. The myristoylated PKC*ζ* pseudosubstrate inhibitor (Enzo Life Sciences) and calphostin C (an inhibitor of conventional and novel PKC isoforms, but not PKC*ζ*) were obtained from EMD Millipore. Other kinase inhibitors and reagents were from Sigma Chemical Co. (St. Louis, MO), and fluorescence-conjugated antibodies and respective isotype control mAbs were from BD Biosciences PharMingen (San Diego, CA), unless otherwise specified.

### Preparation of mouse platelets and neutrophils

Mouse blood platelets were prepared as described previously [16]. Washed platelets were resuspended in Tyrode's buffer (10 mmol/L 4-(2-hydroxyethyl)-1-piperazineethanesulfonic acid, 12 mmol/L NaHCO_3_, 137 mmol/L NaCl, 2.7 mmol/L KCl, 0.42 mmol/L NaH_2_PO_4_, and 5.5 mmol/L glucose, pH 7.4) containing 0.1% BSA. Platelet count was adjusted to 2×10^8^/ml.

For preparation of peritoneal neutrophils [16], mice were injected with 2.5 ml of sterile 3% thioglycolate broth solution (Sigma Chemical Co.) by intraperitoneal administration, and 5 hours after injection neutrophils were harvested and purified using a two-layer Percoll gradient (80% over 64%). Cytospin preparations stained with Giemsa revealed that >95% of the cells were neutrophils. Cell viability (>95%) was assessed using trypan blue exclusion. Isolated neutrophils were resuspended in RPMI 1640 containing 0.5% (w/v) low-endotoxin BSA.

### Neutrophil adhesion to surface-adherent activated platelets

Washed platelets (∼1.5×10^7^ in 100 ul PBS) isolated from WT or CD40L^−/−^ mice were loaded into 96-well microtiter plates that were precoated overnight with 100 ug/ml fibrinogen (Enzyme Research Laboratories). Platelets were allowed to settle, adhere, and spread onto the surface of the fibrinogen-coated wells for 45 minutes and then stimulated with thrombin (0.2 U/ml) for 5 minutes to activate platelets. After that, unbound platelets were removed by washing with RPMI 1640/0.1% BSA. To avoid CD40L shedding from activated platelets, the bound platelets were fixed in 2% paraformaldehyde (PFA) for 15 min at room temperature followed by washing in PBS. The surface expression of CD40L on thrombin-activated platelets was determined by flow cytometry as we described previously [16]. Neutrophils (1.5×10^5^ in 100 ul of RPMI 1640/0.1%BSA) isolated from WT, CD40^−/−^, or Mac-1^−/−^ mice were loaded with 1.0 umol/L calcein AM (Molecular Probes) for 20 minutes, washed twice, and then added to each platelet-coated microtiter well for 60 min. To determine the effects of CD40L on neutrophil adhesion, the calcein AM-loaded neutrophils were incubated without or with rm-CD40L(100 ng/ml) for 60 min. Neutrophil adhesion was assessed by a fluorescence microplate reader (Bio-Tek Instruments, Inc.). The fluorescence signal of neutrophils before washing was measured and served as a measure of total cell number. After washing three times with PBS to remove non-adherent cells, the fluorescence signal was measured again. Data are expressed as the percentage of adherent cells in total cells by calculating the ratio of the fluorescence of calcein AM–loaded cells in each well before and after washing essentially as described previously [18].

### Neutrophil transmigration through surface-adherent activated platelets

Washed mouse platelets (∼2.5×10^7^) were bound transwell membranes (diameter: 6.5 mm; pore size: 3 um; Corning Inc.) that were pre-coated overnight with fibrinogen (100 ug/ml). After incubation for 30 min at 37°C, the transwells were centrifuged at 200 *g* for 2 min and then washed. Bound platelets were activated with thrombin (0.2 U/ml) for 5 min. Calcein AM-loaded neutrophils (5×10^5^) were added to the upper chamber in 200 µl RPMI1640/0.1%BSA. Neutrophil chemotaxis was stimulated by adding the chemoattractant fMLP (1 µM, Sigma) into the lower chamber. A 1:20 dilution of input neutrophils added to a well that contained medium alone, but without a transwell insert, served as a measure of the number of input cells. After incubation for 60 min at 37°C, the transwell inserts were removed, and the transmigrated cells were quantified as the percentage of total cells by measuring the fluorescence of calcein AM–loaded neutrophils, as described [18].

### Measurement of integrin Mac-1 expression and activation in neutrophils

Flow cytometry was performed to detect Mac-1 expression as described previously [16]. Freshly isolated neutrophils (1×10^6^/ml, in RPMI 1640/0.1% BSA) were incubated with 100 ng/ml rm-CD40L for 60 min at 37°C and then fixed in 2% PFA for 15 min. After washing twice, cells were incubated with 3 μl of PE-conjugated rat anti-mouse CD11b mAb (clone M1/70, against integrin αM chain of Mac-1), or FITC-conjugated rat anti-mouse CD18 mAb (clone M18/2, against integrin β2 chain), or respective isotype control mAb, for 30 minutes at room temperature in the dark. The expression levels of CD11b and CD18 subunits were determined by measuring mean fluorescent intensity (MFI) by a Becton Dickinson FACScalibur with CellQuest software.

Fluresence microscopy was used to visualize Mac-1 activation by examining clustering of CD11b on the cell surface. Neutrophils (2×10^5^ cells in 200 µl of RPMI1640/0.1%BSA) were stimulated without or with rm-CD40L (100 ng/ml) for 60 min at 37°C. After fixation in 2% PFA, samples were washed 3 times in PBS/1%BSA and stained by Alexa Fluor 488–conjugated rat anti-mouse CD11b mAb (M1/70) for 30 min at 4°C. Cytospin slides were made by centrifugation (Cytospin 3; Shandon) at 500 rpm for 3 min. Images were analyzed with confocal microscopy.

### Immunofluorescence

Neutrophils (1×10^6^/mL) were seeded onto gelatin-coated glass coverslips (12 mm diameter) and allowed to adhere for 10 min at 37°C and then stimulated without or with rm-CD40L for the indicated times. The cells were then fixed in 2% PFA for 10 min, washed in PBS. Blocking was performed in PBS containing 10% serum specific to the species of the secondary antibody for 1 hour at room temperature. For detection of Mac-1 activation, neutrophils were then labeled with the the anti-Mac-1 (CD11b) antibody (1∶1000) overnight at 4°C, washed with buffer, incubated for 1 h at room temperature with Alexa Fluor 488-conjugated secondary antibody, washed again, and mounted in 100% glycerol on glass slides. For double immunofluorescence staining, neutrophils were first stained with the anti-Mac-1 (CD11b) antibody (1∶1000) and then with anti-PKCζ antibody (1∶200). Immunoreactions were visualized using Alexa Fluor 488- or Alexa Fluor 555-conjugated secondary antibodies (1∶200; Invitrogen). Images were recorded and analyzed with a fluorescence microscope (Nikon).

### Immunoprecipitation and Western Blotting

Neutrophils (1.0×10^7^/mL) were stimulated without or with rm-CD40L (100 ng/ml) for the indicated times. Cells were washed with ice-cold PBS and lysed in the RIPA buffer (1% Nonidet P-40; 0.25% sodium deoxycholate; 1 mm EGTA; 150 mm NaCl; and 50 mm Tris-HCl, pH 7.5) containing a cocktail of protease inhibitors (Sigma) at room temperature for 10 min. The cell lysates were centrifuged at 14,000×*g* for 10 min at 4°C and the pre-cleared cell lysates were immunoprecipitated overnight at 4°C with the anti-PKCζ antibody. The immunoprecipitates were immobilized using protein A beads for 2 h at 4°C and washed three times with the same lysis buffer containing protease inhibitors. The precipitated proteins were eluted in 40 μl 2× Laemmli sample buffer, boiled for 5 min, and separated using 10% SDS-PAGE. The proteins were transferred to Immobilon-P membranes that were blocked for 1 h in 3% BSA or 5% nonfat milk in Tris-saline buffer with 0.2% Tween 20. Immunoblotting was performed with the anti-Mac-1 (CD11b) antibody (1∶500). The bands were normalized by stripping the blots with IgG elution buffer and reprobing them with anti-PKCζ antibody (1∶800). Non-immunoprecipitated lysates were also analyzed by immunoblotting with the same antibodies. Immunopositive bands of HRP-conjugated secondary antibodies were detected with an ECL system (GE Healthcare) and exposure to ECL Hyperfilm.

### Measurement of neutrophil oxidative burst

Oxidative burst was determined by reactive oxygen species (ROS) production in neutrophils that was measured by a flow cytometric approach using 5-(and-6)-chloromethyl-2′,7′-dichlorodihydrofluorescein diacetate (CM-H2DCFDA) as a fluorescent probe. Briefly, aliquots of neutrophils (5×10^5^ cells in 200 μl PBS/0.1% BSA) were loaded with 5 μmol/L CM-H2DCFDA (Molecular Probes) for 30 minutes at 37°C, and then stimulated with 100 ng/ml rm-CD40L for 30 min at 37°C. Samples were analyzed immediately by flow cytometry. Each assay was performed in quadruplicate. Data are expressed as mean fluorescence intensity in FL-1 channel for CM-H2DCFDA.

### NF-kB p65 nuclear translocation assay

NF-kB p65 nuclear translocation in neutrophils was examined by immunocytochemical method, as previously described [19]. Briefly, freshly isolated neutrophils (from WT, CD40^−/−^, and Mac-1^−/−^ mice) were resuspended in RPMI 1640 containing 0.5% (w/v) low-endotoxin BSA. Aliquots of neutrophils (2×10^5^ cells in 200 μl) were stimulated with rm-CD40L (100 ng/ml) for 60 minutes at 37°C. The cells were then fixed with 2% PFA in PBS (pH 7.4) for 20 min at room temperature, followed by permeabilization with 0.2% Triton X-100. Nonspecificbinding was blocked with 5% normal goat serum (Invitrogen) for 1 h. Then, cells were incubated with rabbit polyclonal anti-human p65 antibody (1∶100, Abcam) overnight at 4°C, followed by incubation with Alexa Fluor 488-conjugated goat anti-rabbit IgG (1∶200, Invitrogen) for 1 h at room temperature. Nuclei were stained with DAPI (Vector Laboratories). Images were taken using a fluorescence microscope (Nikon, Japan) and quantification was performed using Image-Pro Plus software. Results were expressed as percentage of nuclear NF-kB p65 positive cells in total cells counted from eight randomly chosen high-power (×200) fields in each well. Each assay was performed in quadruplicate.

### Statistical analysis

Data are presented as means ± SEM and were determined using either two-tailed t-test analysis or one-way ANOVA followed by Fisher's exact test analysis. P values less than 0.05 were considered statistically significant.

## Results

### 1. Platelet surface CD40L mediates neutrophil firm adhesion and transplatelet migration through interaction with neutrophil CD40

**Figure 1:**
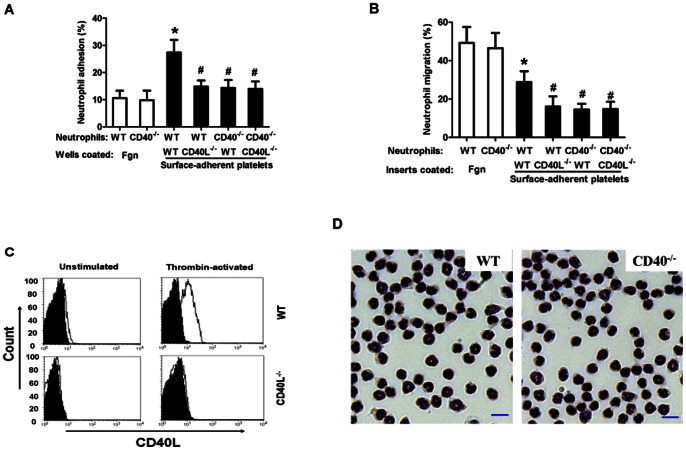
Platelet surface CD40L mediates neutrophil firm adhesion and transmigration via interaction with neutrophil CD40. **A**. Neutrophil firm adhesion to activated surface-adherent platelets. **B**. Neutrophil transmigration across activated, surface-adherent platelets. Microtiter wells or transwell insert membranes were pre-coated with thrombin-activated platelets adherent to fibrinogen (Fgn) or fibrinogen alone. Washed platelets were prepared from WT or CD40L^−/−^ mice. Neutrophils were prepared from WT or CD40^−/−^ mice. Cell adhesion and migration were performed as described in *Methods*. Data obtained from five independent experiments are shown. *P<0.05 versus Bar 1; ^#^P<0.05 versus bar 3. **C**. Flow cytometry showing the surface expression of CD40L in thrombin-activated WT and CD40^−/−^ platelets. **D**. Representative microscopic pictures (×400) showing Giemsa-stained peritoneal neutrophils from WT and CD40^−/−^ mice. Bar = 10 μm.

Platelet activation and leukocyte-platelet interactions represent an important mechanism of leukocyte recruitment at the site of inflammation and vascular injury [2]. Here, we studied neutrophil interactions with activated, surface-adherent platelets as a model for leukocyte recruitment to the sites of injury [18]. CD40L is cryptic in unstimulated platelets but is rapidly presented to the platelet surface after platelet stimulation [15]. Washed platelets were prepared from WT and CD40L^−/−^ mice, and flow cytometry verified the surface expression of CD40L on thrombin-activated WT platelets, but absence of CD40L in CD40L^−/−^ platelets (Fig. 1c). Neutrophils were prepared from WT and CD40^−/−^ mice, and Giemsa stain showed no significant difference in the morphology of WT and CD40^−/−^ neutrophils (Fig. 1d). For adhesion assays, the wells were pre-coated with fibrinogen alone or with fibrinogen to which platelets were bound and then activated and fixed with 2%PFA to prevent CD40L shedding from activated platelets. The firm adhesion to wells coated with fibrinogen alone was similar in WT and CD40^−/−^ neutrophils (Fig. 1a, bars 1, 2). The firm adhesion to wells with adherent platelets was significantly increased in the WT neutrophil → WT platelet group (Fig. 1a, bar 3), however, this increase was almost completely blocked by deletion of either platelet-CD40L or its neutrophil counter receptor CD40 (Fig. 1a, bars 4–6).

For migration assays, the transwell inserts were coated with fibrinogen alone or with fibrinogen to which platelets were bound and then activated. The tranmigration of both WT and CD40^−/−^ neutrophils across adherent platelets was significantly lower than that of tranmigration through fibrinogen alone (Fig. 1b, bars 1, 2). These observations were consistent with previous studies [18], [20], indicating that the surface-adherent platelets acted as barrier to neutrophil chemotaxis [18]. Nevertheless, deletion of either platelet-CD40L or neutrophil-CD40 significantly reduced the neutrophil transmigration across adherent platelets (Fig. 1b, bars 3–6). Together, our data demonstrated that platelet surface CD40L interacting with its counter receptor CD40 expressed in neutrophils significantly promotes neutrophil firm adhesion to and transmigration across surface-adherent platelets.

### 2. sCD40L stimulates CD40-dependent upregulation and activation of the β2 integrin Mac-1 in neutrophils and enhances neutrophil-platelet interactions

**Figure 2:**
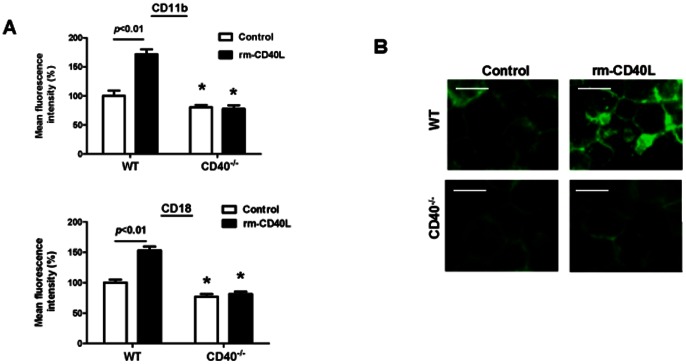
sCD40L stimulates CD40-dependent upregulation and activation of the β2 integrin Mac-1 in neutrophils. **A.** Flow cytometric analysis of Mac-1 (CD11b and CD18) expression in WT and CD40^−/−^ neutrophils stimulated without or with rm-CD40L (100 ng/ml, 60 min). Data obtained from five independent experiments are shown. *P<0.05 versus respective WT control group. **B.** Immunocytochemical staining showing clustering (a sign of Mac-1 activation) of Mac-1 (CD11b) on the cell surface of neutrophils, with robust clustering seen in WT neutrophils stimulated with rm-CD40L, but with weak signals seen in CD40^−/−^ neutrophils. Images were obtained by confocal microscopy. Scale bar  =  10 μm. Similar results are obtained from five independent experiments.

Soluble CD40L (sCD40L) is a truncated form (18 kDa) of the CD40L protein and has been identified as biologically active in vivo and in vitro [21]–[24]. Levels of sCD40L in the blood are elevated in patients with atherosclerosis and acute coronary syndrome, predicting an increased rate of restenosis after vascular interventions [25]. Here, we studied if and how sCD40L directly modulates neutrophil-platelet interactions in vitro. We first examined the effects of sCD40L on neutrophil integrin Mac-1 expression and activation. Flow cytometry showed that stimulation with rm-CD40L (100 ng/ml, 60 min) significantly increased the levels of the surface expression of Mac-1 (both CD11b and CD18 subnits) in WT neutrophils, but it had no effects in CD40^−/−^ neutrophils (Fig. 2a). Immunocytochemical staining showed that stimulation with rm-CD40L (100 ng/ml, 60 min) significantly increased CD11b clustering on the cell-surface of WT neutrophils, indicating an increase in Mac-1 activation (Fig. 2b). However, this increase did not occur in CD40^−/−^ neutrophils (Fig. 2b), suggesting that CD40 is required for sCD40L-induced neutrophil upregulation of Mac-1.

**Figure 3:**
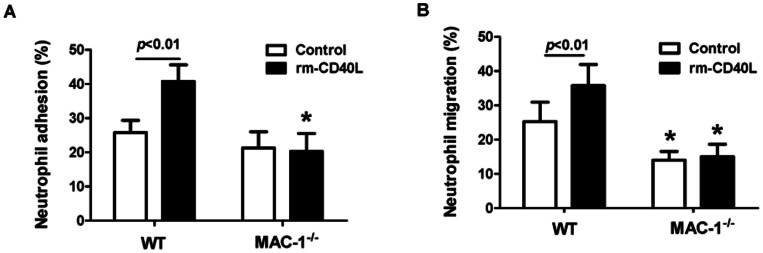
Deficiency of the integrin Mac-1 in neutrophils blocks sCD40L-induced neutrophil firm adhesion and transplatelet migration. **A.** Comparison of WT versus Mac-1^−/−^ neutrophil firm adhesion to activated, surface-adherent platelets. **B**. Comparison of WT versus Mac-1^−/−^ neutrophil transmigration across activated, surface-adherent platelets. Neutrophils were stimulated without and with rm-CD40L (100 ng/ml, 60 min). Data obtained from five independent experiments are shown. *P<0.01 versus respective WT control.

Furthermore, we determined if Mac-1 is required for sCD40L-induced neutrophil firm adhesion and transplatelet migration. Consistent with previous studies [20], Mac-1 deficiency slightly, but not significantly, reduced neutrophil firm adhesion to the surface-adherent platelets (Fig. 3a), but significantly reduced neutrophil transmigration across the surface-adherent platelets (Fig. 3b), when compared with respective WT controls. Nevertheless, Mac-1 deficiency almost completely blocked the rm-CD40L-induced increase in both the firm adhesion (Fig. 3a) to and the transmigration (Fig. 3b) across the surface-adherent platelets.

### 3. PKCζ is required for sCD40L-induced Mac-1 activation and neutrophil adhesion

**Figure 4:**
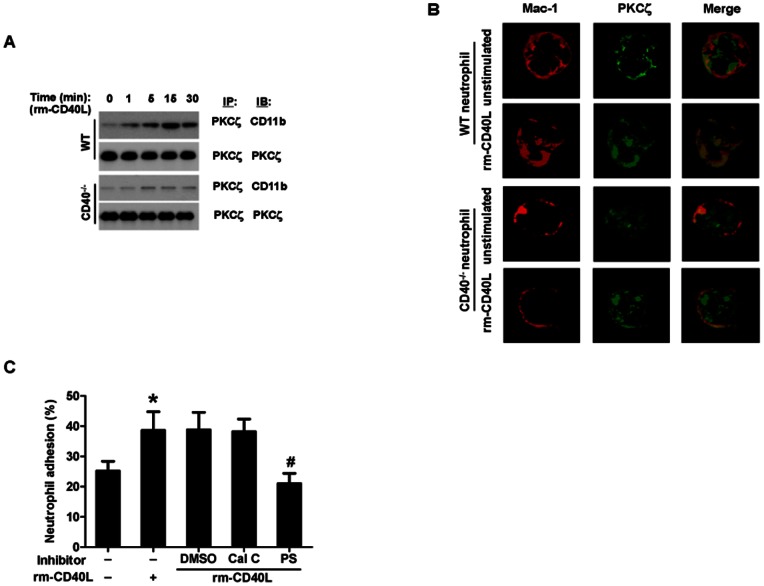
PKCζ is required for sCD40L-induced Mac-1 activation and neutrophil adhesion. **A.** Immunoprecipatation and Western blotting. Neutrophils were stimulated with rm-CD40L (100 ng/ml) for the indicated periods of time. The cell lysates were immunoprecipitated (IP) with anti-PKCζ antibody. Immunoblotting (IB) was carried out using anti-CD11b (Mac-1) antibody. The blots were stripped and reprobed with anti-PKCζ antibody. A set of representative blots from three independent experiemnts is shown. **B.** Double immunofluoresence staining of PKCζ and Mac-1 (CD11b). Stimulation with rm-CD40L (100 ng/ml, 15 min) significantly increased focal surface clustering of Mac-1 and the colocalization PKCζ and Mac-1 in WT but not in CD40^−/−^ neutrophils. **C.** Inhibition of PKCζ activity reduces CD40L-induced neutrophil firm adhesion to activated, surface-adherent platelets. Neutrophils were pretreated (30 min) with a PKC*ζ* pseudosubstrate (PS, 5 μM) or calphostin C (Cal C, 1 μM), an inhibitor of conventional and novel PKC isoforms. Dimethyl sulfoxide (DMSO) was used as a solvent control. Data obtained from five independent experiments are shown. *P<0.05 versus Bar 1; ^#^P<0.05 versus bar 2.

The atypical protein kinase C zeta (PKCζ) plays a critical role in human neutrophil Mac-1 activation and adhesion [26]. In this study, we first examined the role of PKCζ in CD40L-stimulated Mac-1 activation. By immunoprecipitation of neutrophil lysates using an anti-PKCζ antibody, we examined the association of PKCζ with Mac-1 (CD11b). The baseline association between CD11b and PKCζ was low in unstimulated neutrophils. Stimulation with rm-CD40L (100 ng/ml) time-dependently increased the association at 5, 15, and 30 minutes in WT neutrophils, but this stimulatory effect was blocked in CD40^−/−^ neutrophils (Fig. 4a). We further investigated this finding by double immunofluoresence staining. The results showed that stimulation with rm-CD40L (100 ng/ml, 15 min) significantly increased the degree of colocalization of PKCζ with Mac-1 in WT neutrophils, but not in CD40^−/−^ neutrophils (Fig. 4b).

Subsequently, we examined the role of PKCζ in CD40L-stimulated neutrophil adhesion to surface-adherent platelets by two different pharmacological inhibitors: the atypical PKCζ pseudosubstrate (myristoylated), a cell permeable PKCζ specific inhibitor, and calphostin C, an inhibitor of conventional and novel PKC isoforms but without effect on PKCζ. This PKCζ inhibitor has been shown to specifically block the function of PKCζ [27], [28]. The results showed that the PKCζ inhibitor significantly inhibited rm-CD40L-stimulated neutrophil adhesion to surface-adherent platelets (Fig. 4c). In contrast, neither calphostin C (Fig. 4c) nor a control myristoylated peptide directed against PKCα and PKCβ (data not shown) had any effect on neutrophil adhesion.

### 4. CD40, but not Mac-1, is required for sCD40L-induced neutrophil oxidative burst

**Figure 5:**
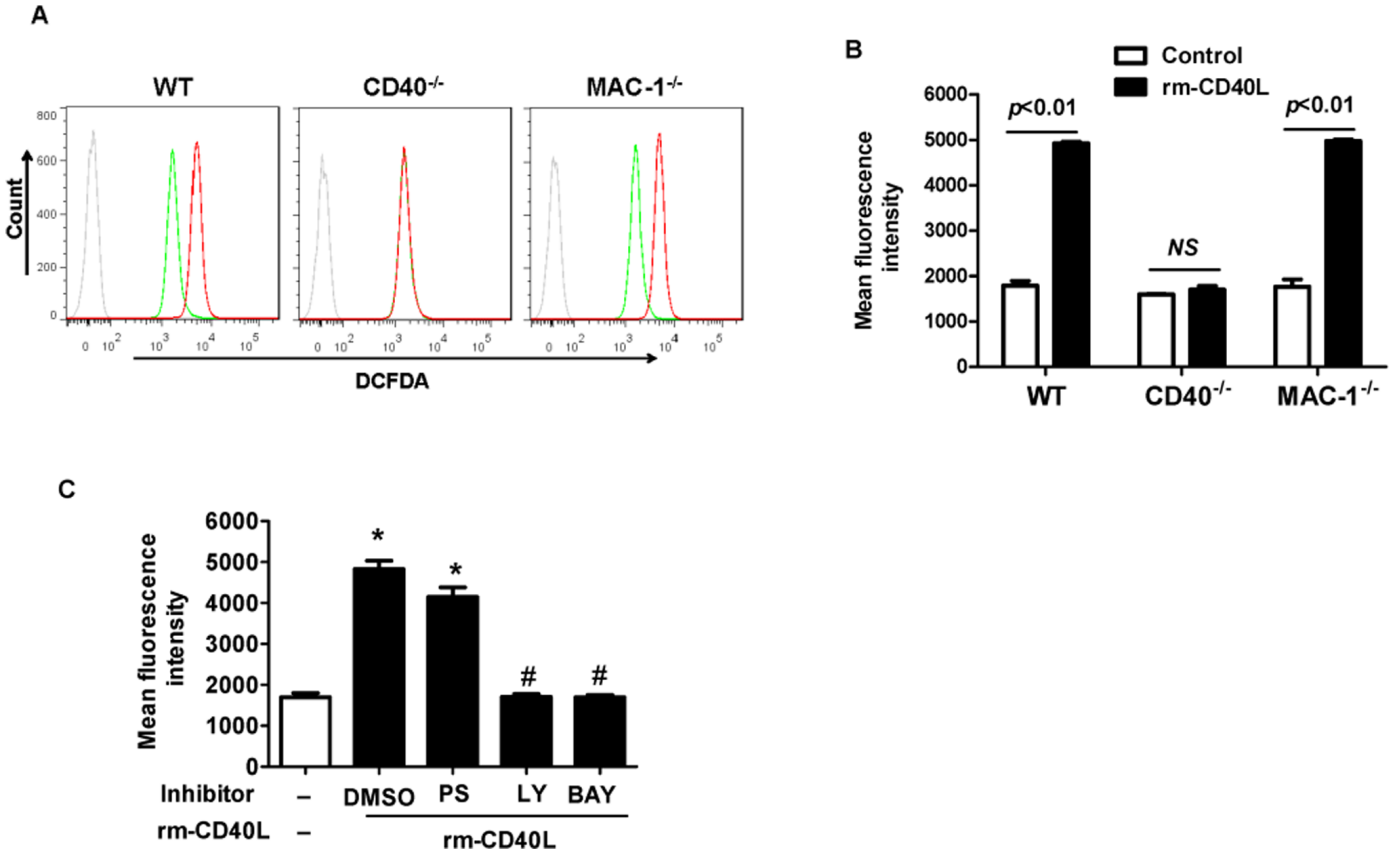
Effects of CD40, Mac-1, and different protein kinase inhibitors on sCD40L- stimulated neutrophil oxidative burst. **A & B:** CD40, but not Mac-1, is required for sCD40L- stimulated neutrophil oxidative burst. **A**. Representative flow cytometry plots showing ROS generation in WT, CD40^−/−^, and Mac-1^−/−^ neutrophils using CM-H2DCFDA as a fluorescent probe (dashed gray line: without the probe as a control). Neutrophils were stimulated without (green line) or with (red line) rm-CD40L (100 ng/ml) for 30 min. **B**. Quantitation of ROS generation in the indicated groups of neutrophils. Data obtained from five independent experiments are shown. NS  =  not significant. **C**. Effect of the indicated protein kinase inhibitors on sCD40L-stimulated neutrophil oxidative burst. WT neutrophils were stimulated without or with rm-CD40L (100 ng/ml) for 30 min in the presence of PKC*ζ* pseudosubstrate (PS, 5 μM), PI3-kinase (LY294002,10 µM), or NF-κB (BAY11-7082, 10 µM) inhibitors, or dimethyl sulfoxide (DMSO) as a solvent control. Data obtained from five independent experiments are shown. *P<0.01 versus bar 1, and #P<0.01 versus bar 2.

Oxidative burst represents as one of the most important aspects of neutrophil functions in inflammation. We measured oxidative burst by flow cytometry using CM-H2DCFDA as a fluorescent probe in neutrophils isolated from WT, CD40 ^−/−^, and MAC-1 ^−/−^ mice. Our data showed that rm-CD40L (100 ng/ml, 30 min) significantly enhanced neutrophil CM-H2DCFDA fluorescence, with comparable levels in WT and MAC-1 ^−/−^ neutrophils (Fig. 5a, 5b), but it had no effect in CD40-deficient neutrophils (Fig. 5a, 5b). Furthermore, our data showed that this rm-CD40L-timulated oxidative burst in WT neutrophils was completely inhibited with PI3-kinase (LY294002, 10 µmol/L) or NF-κB (BAY11-7082, 10 µmol/L) inhibitors, but is was only slightly inhibited with the PKC*ζ* pseudosubstrate inhibitor (PS, 5 µmol/L) (Fig. 5c).

### 5. sCD40L stimulates neutrophil oxidative burst via CD40-dependent activation of PI3K/NF-KB, but independent of Mac-1 and PKCζ

**Figure 6:**
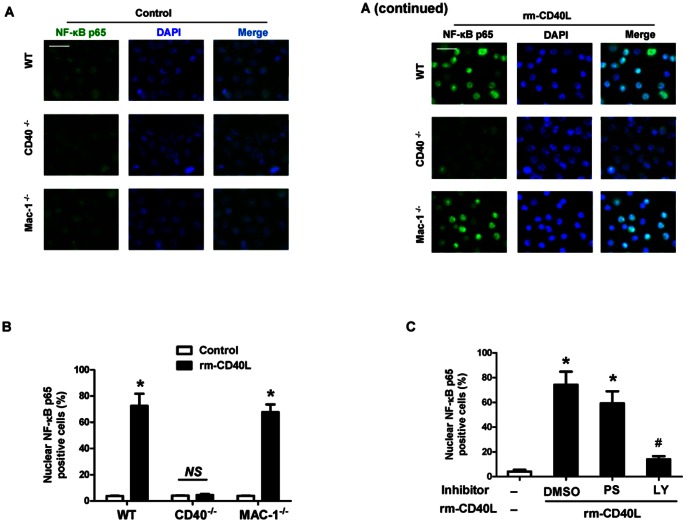
sCD40L induces CD40-dependent NF-kB p65 nuclear translocation in a PI3K- dependent manner. **A.** Representative images of Immunofluorescence staining showing NF-kB p65 (green) nuclear translocation in WT, CD40^−/−^, and Mac-1^−/−^ neutrophils stimulated without or with rm-CD40L (100 ng/ml, 60 min). Cell nuclei were detected by DAPI (blue). Scale bars: 20 μm. **B.** Quantitation of NF-kB p65 nuclear translocation in the indicated groups. Results are expressed as the percentage of the p65 nuclei-positively stained cells to the total cells. **C.** Effect of the indicated protein kinase inhibitors on sCD40L-stimulated NF-kB p65 *nuclear translocation* in neutrophils. WT neutrophils were stimulated without or with rm-CD40L (100 ng/ml) for 30 min in the presence of PKC*ζ* pseudosubstrate (PS, 5 μM), PI3-kinase (LY294002,10 µM), or NF-κB (BAY11-7082, 10 µM) inhibitors, or DMSO as a solvent control. Data obtained from five independent experiments are shown. *P<0.01 versus bar 1, and #P<0.01 versus bar 2.

NF-κB is a major transcription factor that regulates genes responsible for inflammatory and oxidative responses in a variety of cells including neutrophils [29]. By immunofluorescence staining of the translocation of cytoplasmic NF-kB p65 to the nucleus, our data indicated that stimulation with rm-CD40L (100 ng/ml, 60 min) significantly enhanced NF-κB p65 nuclear translocation, with comparable levels in wild-type and Mac-1-deficient neutrophils (Fig. 6a, 6b). In contrast, rm-CD40L failed to stimulate NF-κB p65 nuclear translocation in CD40^−/−^ neutrophils (Fig. 6a, 6b). Moreover, the NF-κB p65 nuclear translocation induced by rm-CD40L was almost completely abrogated with PI3-kinase (LY294002,10 µmol/L), but it is was only slightly inhibited with the PKC*ζ* pseudosubstrate inhibitor (PS, 5 µmol/L) (Fig. 6c).

## Discussion

The pivotal role of CD40L/CD40 in the pathogenesis of atherosclerosis and restenosis is now widely accepted [30], [31]. Disrupting the CD40L-CD40 co-stimulatory pathway reduces atherosclerosis and induces a stable atherosclerotic plaque phenotype [31]. Therefore, inhibition of the CD40L-CD40 pathway is an attractive therapeutic target to reduce clinical complications of atherosclerosis. Our laboratory has focused on investigating the role of CD40L/CD40 in the vascular response to injury. We have demonstrated that either antibody blockade of CD40L or genetic deletion of its receptor CD40 reduces early leukocyte (neutrophil and monocyte) accumulation in the injured arteries and limits eventual neointima formation after vascular injury in mice [16], [19], [32]. Nevertheless, the exact involvement of CD40L/CD40 in neutrophil recruitment and function remains elusive. The adhesion of leukocytes to platelets deposited at the site of injury represents an important mechanism of leukocyte recruitment after vascular injury [2], [3]. This adhesive interaction between platelets and leukocytes is mediated by multiple ligand-receptor pairs [33]. One important pair involves the interaction of P-selectin expressed on the activated platelet surface with P-selectin glycoprotein ligand-1 (PSGL-1), its counter receptor, expressed on leukocytes [18], [34]. Since the presence of CD40L on the activated platelet surface and its counter receptor CD40 in neutrophils is well defined, it was important that we first studied neutrophil interactions with activated, surface-adherent platelets as a model for leukocyte recruitment to the sites of vascular injury. In resting platelets, CD40L resides in intraplatelet stores, but it translocates rapidly to the platelet surface within a few minutes after stimulation [15]. To characterize the role of platelet surface CD40L, platelets were activated with thrombin and then fixed with 2%PFA to prevent CD40L shedding from activated platelets. The present data demonstrate that platelet surface CD40L contributes to neutrophil firm adhesion to and transmigration across activated surface-adherent platelets through interaction with its counter receptor CD40 in neutrophils, as the platelet-dependent neutrophil adhesion and migration is equally attenuated by absence of either platelet CD40L or neutrophil CD40. The findings may also provide a potential explanation for the mechanisms by which platelet deposition at the site of injury contributes to neutrophil recruitment after vascular injury.

Activated platelets are also present in the circulating blood of patients with cardiovascular disease. It has been reported that more than 95% of circulating sCD40L is derived from activated platelets [14], [15], thus implicating activated platelets as a major contributor in a variety of inflammatory diseases in which sCD40L is involved. Indeed, elevated plasma levels of sCD40L are present in patients with cardiovascular disease and predict an increased rate for restenosis and acute ischemic events in patients [25]. A recent study has provided the direct evidence that sCD40L increased neointimal formation after carotid wire injury in mice [17]. Together, these findings prompted us to investigate if and how sCD40L contributes to neutrophil-platelet interactions. The present data demonstrate that recombinant soluble CD40L (rm-CD40L) strongly activates the integrin Mac-1 in wild-type neutrophils whereas has no effect in CD40^−/−^ neutrophils. Importantly, we demonstrate that PKCζ is critically required for rm-CD40L-mediated activation of the integrin Mac-1 and adhesive function of neutrophils. Activation of the leukocyte-specific integrin Mac-1 is known to be critical for mediating leukocyte firm adhesion and recruitment in vitro and in vivo [18], [20]. Protein kinase C (PKC) isoforms are classified into three major classes: conventional, novel, and atypical. Neutrophils are known to express several different PKC isoforms, including three conventional isoforms (α, βI, βII), one novel isoform δ, and one atypical isoform ζ [35]. The atypical PKCζ has been implicated in Mac-1 activation, neutrophil adhesion and chemotaxis [26]–[28]. The present data show that rm-CD40L strongly stimulates the colocalization of the integrin Mac-1 with PKCζ in wild-type neutrophils, but this stimulatory effect is blocked in CD40^−/−^ neutrophils. Blocking PKCζ completely inhibited rm-CD40L-induced neutrophil adhesive function. Taken together, these findings demonstrate that soluble CD40L can activate the leukocyte-specific integrin Mac-1 in neutrophils and thus further enhances platelet-dependent neutrophil adhesion and transmigration, in a PKCζ-dependent manner. The findings may also provide a potential explanation for the mechanisms by which circulating elevated sCD40L contributes indirectly to neutrophil recruitment after vascular injury, through a CD40- and PKCζ-dependent activation of the integrin Mac-1 in neutrophils.

Oxidative stress is widely considered as a common signaling mechanism of the vascular response to injury. Oxidative stress is enhanced in patients with cardiovascular disease, which have been associated with elevated levels of plasma soluble CD40L [36]. We have previously reported that recombinant human CD40L promotes oxidative burst in human neutrophils via a PI3-kinase dependent signaling pathway [16]. However, it is unknown whether this effect is mediated mainly through its classic CD40 receptor and/or leukocyte-specific integrin Mac-1. To address this important mechanistic question, in the present study, we used neutrophils isolated from CD40- and Mac-1-deficient mice. The present data clearly demonstrated that rm-CD40L significantly stimulates ROS generation with a comparable level in wild-type and Mac-1−/− neutrophils, but has no effect in CD40^−/−^ neutrophils. This finding provides the first direct evidence that sCD40L-stimulated ROS generation in neutrophils is CD40-dependent, but Mac-1-independent. The present data reveal that sCD40L-simulated ROS generation is regulated via a CD40-dependent activation of NF-kB but is PKCζ-independent in neutrophuils. We demonstrated that rm-CD40L significantly stimulates NF-kB p65 nuclear translocation, a key step in activation of the NF-kB pathway in neutrophils [37], with a comparable level in wild-type and Mac-1^−/−^ neutrophils, but has no effect in CD40^−/−^ neutrophils. This finding indicates that sCD40L-stimulated NF-kB nuclear translocation in neutrophils is CD40-dependent, but Mac-1-independent. Furthermore, inhibition of NF-kB activity almost completely abolished sCD40L-stimulated NF-kB p65 nuclear translocation and ROS generation, in contrast, inhibition of PKCζ activity has only slight effects. Together, our findings suggest that sCD40L/CD40 contributes to neutrophil-platelet interactions and neutrophil oxidative burst through different mechanisms.

In summary, the results of the present study suggest that CD40L contributes to neutrophil firm adhesion and transmigration across activated surface-adherent platelets, possibly through two potential mechanisms. One involves the direct interaction of ligand-receptor (CD40L-CD40), i.e., platelet surface CD40L interaction with neutrophil CD40; another involves an indirect mechanism, i.e. soluble CD40L (sCD40L) stimulates activation of the integrin Mac-1 in neutrophils and thereby further promotes neutrophil adhesion and migration. Activation of Mac-1 is known to be critical for mediating neutrophil adhesion and migration. Moreover, our data reveal that PKCζ is critically required for sCD40L-induced Mac-1 activation and neutrophil adhesive function, but it is weakly associated with sCD40L-induced neutrophil oxidative stress. These findings may contribute to a better understanding of the underlying mechanisms by which sCD40L/CD40 pathway contributes to inflammation and vascular diseases.
